# Unusual thoracic tumor in a teenager: A rare occurrence

**DOI:** 10.5339/qmj.2022.12

**Published:** 2022-03-01

**Authors:** Tasnim Muhamad, Khairil Amir Sayuti, Noor Hasnita Ismail Mokhtar, Nor Hayati Yunus, Noor Syafawati Ismail

**Affiliations:** ^1^Department of Radiology, School of Medical Sciences, Universiti Sains Malaysia, Jalan Raja Perempuan Zainab II, 16150 Kubang Kerian, Kelantan, Malaysia E-mail: khairilamirsayuti@yahoo.com; ^2^Hospital Universiti Sains Malaysia, Jalan Raja Perempuan Zainab II, 16150 Kubang Kerian, Kelantan, Malaysia E-mail: khairilamirsayuti@yahoo.com; ^3^Department of Radiology, Hospital Raja Perempuan Zainab II, 15586 Kota Bharu, Kelantan, Malaysia; ^4^Department of Pathology, Hospital Raja Perempuan Zainab II, 15586 Kota Bharu, Kelantan, Malaysia

**Keywords:** neoplasms, thoracic neoplasm, lung neoplasm, pulmonary blastoma

## Abstract

Pleuropulmonary blastoma (PPB) is a rare malignant lung tumor in the pediatric population and occurs mainly in young children. Its clinical presentation is usually nonspecific. We report a rare occurrence of this tumor in a 15-year-old girl, who presented with symptoms mimicking respiratory tract infection and was nonresponsive to the initial treatment. Imaging investigations revealed a large solid lesion in the left hemithorax with a mass effect on the adjacent structures. Biopsy demonstrated primitive cells with blastematous appearances, and the stroma cells were positive for vimentin and desmin, consistent with PPB. Unfortunately, she died from neutropenic sepsis while undergoing chemotherapy. This report highlights the epidemiology of PPB, its imaging and histopathological features, overview of prognosis, and clinical management.

## Introduction

Pleuropulmonary blastoma (PPB) is one of the primitive tumors and occurs almost exclusively in children, especially in the first decade of life. Patients normally present with pneumonia and respiratory distress.^1^ Radiological findings include a large thoracic mass with heterogeneous low attenuation, associated with pleural effusion, and absence of chest wall involvement.^2^ Most reports described right hemithorax involvement with mass effect on the mediastinum.^2,3^ Histologically, it is divided into types 1, 2, and 3. Type 1 shows a cystic thin-walled structure. Type 2 has mixed solid cystic components, with nodule and plaque-like thickening and solid polypoid regions extending into the cysts. Type 3 is a completely solid tumor. The solid regions in type 2 and 3 PPB consist of mixed blastematous and sarcomatous elements and cartilaginous nodules.^4^ Immunohistochemical study typically shows reactivity of the tumor cells to vimentin and reactivity of rhabdomyoblastic cells to desmin antibody.^1^

The prognosis varies depending on the tumor type. Type 1 has a good prognosis, whereas the survival rates of types 2 and 3 vary, with higher chances of recurrence.^5^ Patients with mediastinal and pleural involvement also have significantly poorer survival outcomes.^3^ Metastasis most commonly involves the skeletal and central nervous systems. Type 2 and 3 PPBs have shown strong relations with cerebral metastasis.^6^ A relationship between PPB and DICER1 syndrome was also found in 2009. DICER1 syndrome is an autosomal dominant disorder because of mutation of the *DICER1* gene, which then predisposes individuals to multiple cancers such as cystic nephroma, rhabdomyosarcoma, Wilm's tumor, and differentiated thyroid carcinoma.^5^

Treatments include neoadjuvant chemotherapy, chemotherapy, and surgical resection depending on the tumor types and other local factors.^5^ Given the rarity of PPB, evidence on the best treatment is still inadequate. Nevertheless, complete tumor resection was found to improve prognosis in certain patients.^7^ Preoperative needle aspiration of pleural effusion or pneumothorax is recommended for those with respiratory distress. Chest tube insertion should be avoided if possible because of the risk of contaminating the pleura. For type 1 PPB, if there are numerous cysts at various locations, only the largest cyst should be removed, preferably through a thoracotomy approach. Total pneumonectomy and thoracoscopic procedure are discouraged. There is no consensus on the use of adjuvant chemotherapy for type 1 PPB. However, it can be considered to avoid relapse or progression into type 2 or 3. Radiotherapy is not recommended. For type 2 and 3 PPB, chemotherapy is mandatory for those with an opportunity for cure. Surgical resection is offered after neoadjuvant chemotherapy if complete macroscopic clearance can be obtained with minimal postoperative complication and for cases without metastatic disease. Immediate debulking surgery is reserved for patients with fatal conditions. Radiotherapy is suggested in cases with residual viable tumors after chemotherapy and in second-look surgery.^8^

## Case Report

A 15-year-old girl presented to an outpatient clinic with fever, cough, and lethargy for 3 days. She had no underlying medical illness. On examination, she was febrile (38.3°C), mildly tachypneic with good oxygen saturation, and hemodynamically stable. Breath sounds were diminished in the left lung, with crepitation at its base. Septic parameters were slightly elevated with total white cell count of 17.6 × 10^3^/μL and platelet count of 487 × 10^3^/μL, which led to the commencement of oral antibiotics. In view of the nonimproving symptoms, she was brought to the casualty department several days later for further investigation.

**Figure 1.**
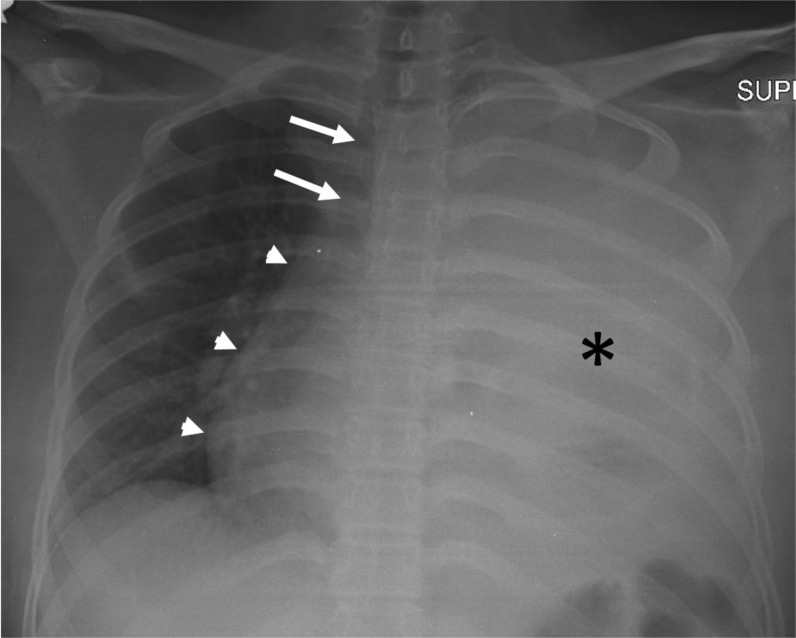
Chest radiography demonstrates opacity occupying the left hemithorax (*) causing obliteration of the left heart border, aortic knuckle, and left hemidiaphgram. The mediastinum (arrowheads) and trachea (arrows) shifted to the right.

**Figure 2.**
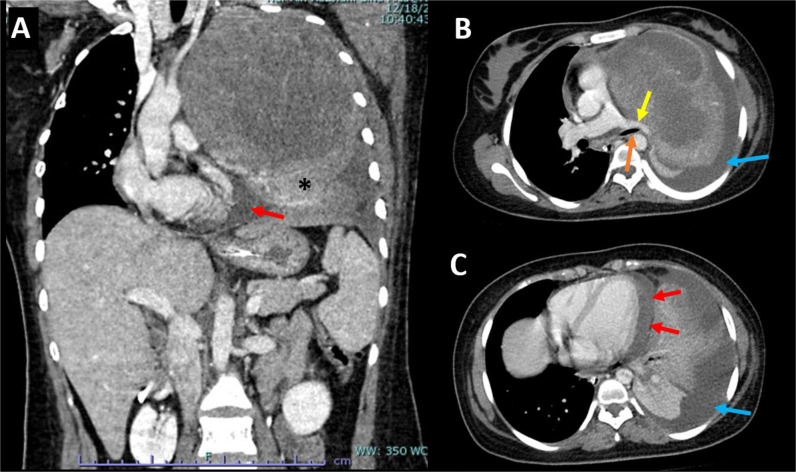
Contrast-enhanced computed tomography of the thorax in coronal (A) and axial (B and C) planes shows a large enhancing mass with central necrosis occupying the left hemithorax. It exerts mass effect, causing contralateral mediastinal shift, collapse, inferior displacement of left lower lobe (*), and narrowing of the left main bronchus (orange arrow) and the left pulmonary artery (yellow arrow). Pericardial (red arrows) and left pleural effusions (blue arrows) are present. The right hemithorax and upper abdominal organs are unremarkable.

Chest radiography showed homogenous opacification of nearly the entire left hemithorax, obscuring the left heart border, aortic knuckle, and left hemidiaphragm. The mediastinum shifted contralaterally. The right lung field showed no focal lesion (Figure 1). Computed tomography (CT) of the thorax revealed a large low-attenuation enhancing mass within the left thoracic cavity, measuring 11 × 13 × 16 cm. The mass was thought to originate from the left upper lobe, causing the collapse of the whole left lung. No intralesional hemorrhage or calcification was observed. Nonenhanced areas are seen within the mass, which likely represent necrosis. The mediastinum shifted to the right. The distal left main bronchus was narrowed, with tapering and occlusion of its segmental bronchi. The pulmonary trunk and left pulmonary artery were also narrowed but remained patent. There was no fat plane between the mass and pericardium, with evidence of pericardial and left pleural effusions. The chest wall was not affected. There was no evidence of mediastinal lymphadenopathy or liver lesions. These findings were suggestive of an aggressive left lung tumor, and CT-guided tissue biopsy was subsequently performed (Figure 2).

**Figure 3.**
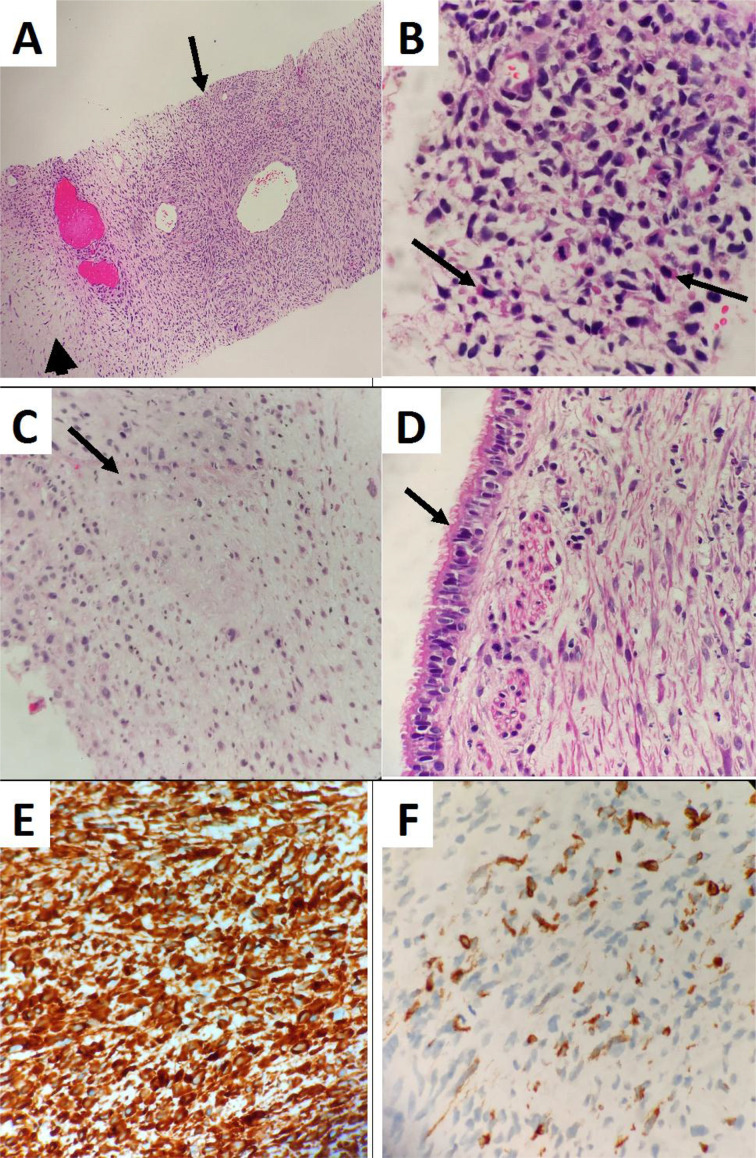
Photomicrographs of the biopsied thoracic mass: (A) Tumor cells arranged in hypocellular (arrow head) and hypercellular areas (arrow) (hematoxylin and eosin [H&E], × 100). (B) The tumor cells are pleomorphic, spindled to ovoid displaying hyperchromatic nuclei with variable amounts of eosinophilic cytoplasm, and frequent mitotic figures. Few isolated pleomorphic rhabdoid cells (arrows) with intense eosinophilic cytoplasm are observed (H&E, × 600). (C) Area of immature cartilage (arrow) (H&E, × 600). (D) Sub-epithelial collection of stromal cells with overlying native ciliated columnar epithelium (arrow) (H&E, × 600). The tumor cells show diffuse cytoplasmic positivity for vimentin (E, IHC, × 600) and focal moderate cytoplasmic staining for desmin (F, IHC, × 600).

The tissue biopsy measured 15 and 18 mm. Microscopic examination revealed tumor cells configured in hypocellular and hypercellular patterns with an area of necrosis. The hypercellular area displayed primitive cells with blastematous appearances. The shape ranged from spindled to ovoid, with focal pleomorphic to rhabdoid cells having intense eosinophilic cytoplasm. Subepithelial mesenchyme with the collection of stromal cells and overlying native ciliated columnar epithelium were observed. A focal immature chondroid element was identified. In this case without an overt rhabdomyoblastic component, entities such as monophasic synovial sarcoma, malignant teratoma, and undifferentiated sarcoma were considered in the differential diagnosis and excluded. Immunohistochemistry of the stromal cells was strongly positive for vimentin and focal moderately positive for desmin. They were negative for EMA, pan-cytokeratin (AE1/AE3), S100, smooth muscle actin, and myogenin. CD99 shows focal nonspecific positivity, while TTF-1 highlights the presence of native respiratory epithelium and alveolar pneumocytes. These findings were consistent with pleuropulmonary blastoma (Figure 3). Another consultant pathologist who specialized in lung pathology also reviewed the biopsy and concurred with this diagnosis.

**Figure 4.**
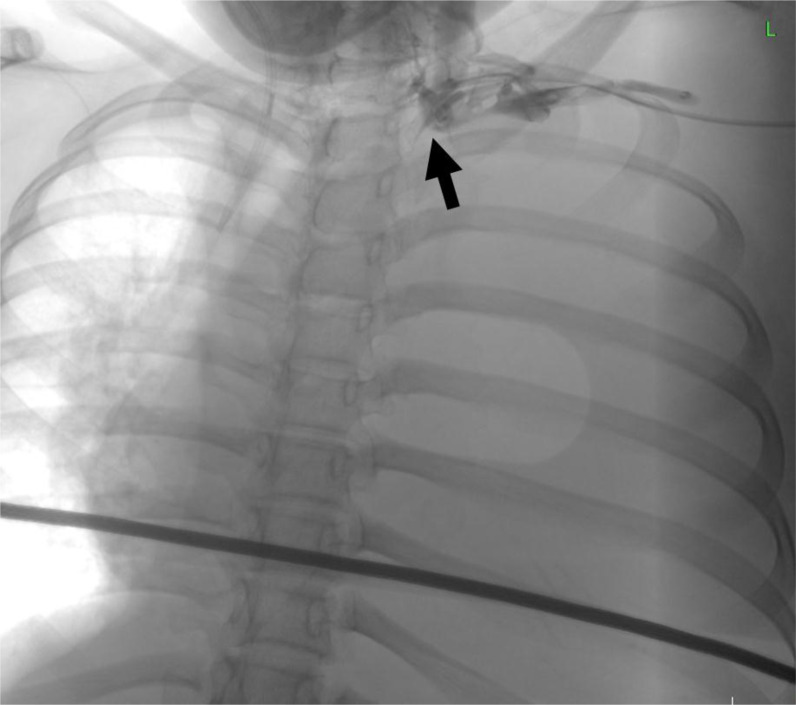
Central venography from the left approach demonstrates complete occlusion at the confluence of the left internal jugular and left subclavian veins (arrow). No contrast flow is seen in the left brachiocephalic vein. Collateral vessels are present.

Neoadjuvant chemotherapy was planned to reduce the tumor size before the surgical resection. However, the central venography for attempted central line insertion has shown complete occlusion at the confluence of the left internal jugular and subclavian veins with the formation of collateral vessels (Figure 4). Eventually, she managed to undergo chemotherapy through the left femoral venous access; unfortunately, she died from neutropenic sepsis while undergoing the treatment.

## Discussion

PPB is a rare thoracic tumor of young children with a recorded incidence of 20–50 cases annually in the USA.^5^ The worldwide registry on tumor incidence is unavailable. The age of presentation is usually less than 1 year in types 1 and 2 and 4–5 years in type 3, with equal gender predilection. Occurrence beyond the first decade of life is considered unusual.^1^ The disease was diagnosed in our patient at a very rare age. Only a few PPB cases were recorded during adolescence, and it was even fewer in the middle age.^9–12^ Despite the association of the *DICER1* syndrome with PBB, our patient was not tested for *DICER1* gene mutations, and she had no family history of cancer. However, many patients with PPB were found to have a negative family history for PPB-associated cancer.^13^ Therefore, screening for *DICER1* carriers among her siblings or first-degree relatives may be beneficial, as it can potentially lead to the earlier detection of related cancers or type 1 PBB before progression to types 2 and 3.

With plain radiography, PPB may result in the complete opacification of the hemithorax with associated mass effect. In CT, type 1 tumor appears as air-filled single or multicystic lesion. Type 2 lesions have air- or fluid-filled cavities with possible air-fluid levels along with solid internal nodules. Type 3 lesions demonstrate low-attenuation solid components with heterogeneous or homogeneous enhancement post contrast, as seen in our case. Areas of central necrosis were also present in our case, which was likely due to the rapid tumor growth. In magnetic resonance imaging, type 3 tumors are heterogeneous in appearance with areas of internal hemorrhage, enhancement regions, and restricted diffusion.^4^ Previous case reports have described variable features of PPB such as cystic, solid cystic, and purely solid.^2,9^ Unlike our patient, PPB was found in the right hemithorax in all previously reported cases. Similar to our case, the tumor is usually associated with contralateral mediastinal shift and pleural effusion. Chest wall invasion is typically absent.^2,3^

The differential diagnosis for primary lung tumors in children includes neuroblastoma, Ewing sarcoma, Askin tumor, and rhabdomyosarcoma.^2,4^ However, these tumors invade the chest wall, a feature not found in our case. Our patient's tumor was also predominantly solid. Therefore, diseases such as cystic pulmonary airway malformation, bronchogenic cysts, and extralobar pulmonary sequestration were not considered alternative diagnoses in CT.^3,4^

The solid tumor in our case was consistent with type 3 PPB, and the immunochemical testing was positive for desmin and vimentin, which further supported the diagnosis. By contrast, most of the previously reported PPB cases among adolescents were types 1 and 2.^9,10,12^ Since PPB is a rare tumor, the histology should be reviewed by pathologists with expertise in pediatric tumors, which was conducted in our case.^8^ In general, type 2 and 3 PPBs carry a worse prognosis than type 1. Type 3 tumor, which was seen in our patient, has the poorest prognosis with a 5-year disease-free survival of 37% compared with 59% in type 2.^12^ Although our CT did not show evidence of chest wall involvement and metastases to the lymph node or liver, the tumor was very large. The loss of fat plane with the presence of pleural and pericardial effusion may suggest reactive inflammatory changes or tumor infiltration into the pleura and pericardium. Approximately 11% of type 3 PPB have metastasized at the time of presentation.^12^ Since our patient did not have neurological or skeletal symptoms or signs, she was not subjected to brain, spine, or bone imaging. Similar CT findings were found in an 18-year-old man who died from respiratory dysfunction, which was likely due to airway obstruction before the treatment was started.^9^

Since our patient was symptomatically stable and did not have clear evidence of metastasis, she underwent neoadjuvant chemotherapy to shrink the tumor before resection. Unfortunately, the patient developed neutropenic sepsis during the early commencement of her chemotherapy and subsequently succumbed to this complication. In a previously reported PPB case, a 14-year-old boy with nearly half the tumor size managed to undergo radiotherapy and chemotherapy and had an overall survival of 27 months before he was lost to follow-up.^9^

## Conclusion

Although PPB is a rare tumor and occurs mostly in young children, it should be also considered in teenagers, especially when there is a large unilateral thoracic mass without chest wall invasion. Clinical presentation is usually nonspecific, but respiratory distress is an expected complication. Tissue diagnosis is mandatory since the treatment modality and prognosis depend on the histological types of PPB, presence of a regional mass effect, and distant metastasis. Family members are encouraged to undergo genetic screening for *DICER1* mutation for the early detection of the associated malignancy.

